# Automated colorimetric determination of recombinant fungal laccase activity in fermentation sarples using syringaldazine as chromogenic substrate

**DOI:** 10.1155/S1463924698000261

**Published:** 1998

**Authors:** K. A. Holm, D. M. Nielsen, J. Eriksen

**Affiliations:** Analytical Biotechnology R & D Enzyme Research Novo Nordisk Research Institute Bagsvaerd DK-2880 Denmark

## Abstract

An automated Cobas Fara method was developed determining the activity of recombinant M. thermophila laccase (rMtL). The chromogenic substrate used was syringaldazine. Under aerobic conditions, rMtL catalyses the oxidation of syringaldazine forming tetrametoxy-azo bis methylene quinone. The developed violet colour was measured kinetically at 530 nm as an expression of the enzyme activity, rMtL is a very sensitive oxidoreductase, therefore many factors had to be carefully controlled in order to get a robust analytical assay. In order to stabilize rMtL, PEG 6000 was added to the enzyme dilution medium. Furthermore, Triton X-I00 was included in the enzyme incubation solution.

The analytical as well as technical conditions have been optimized, resulting in a method with good precision, sensitivity and speed of analysis. The Michaelis-Menten constant, K_m_, was determined to be 22μM syringaldazine. LOQ was determined to be 0.010 Uml^-1^, LOD to be 0.0002 Uml^-1^ The analytical range of the enzyme dilution curve was from 0.01 to 0.044 Uml^-1^ The repeatability was 1.9%, the reproducibility 3.1%. Testing the robustness of the method showed that the most sensible factors in the rMtL analysis in decreasing range were: incubation temperature, concentration of Triton X-I00, molarity and pH of the incubation buffer, and finally the concentration of syringaldazine.


Journal of Automatic Chemistry, Vol. 20, No. 6 (November–December 1998) pp. 199–203

### Automated colorimetric determination of recombinant fungal laccase activity in fermentation samples using syringaldazine as chromogenic substrate

**K. A. Holm, D. M. Nielsen and J. Eriksen**

*Analytical Biotechnology R & D, Enzyme Research, Novo Nordisk Research Institute, DK-2880 Bagsvaerd, Denmark*

*An automated Cobas Fara method was developed determining the activity of recombinant M. thermophila laccase (rMtL). The chromogenic substrate used was syringaldazine. Under aerobic conditions, rMtL catalyses the oxidation of syringaldazine forming tetrametoxy-azo bis methylene quinone. The developed violet colour was measured kinetically at 530 nm as an expression of the enzyme activity. rMtL is a very sensitive oxidoreductase, therefore many factors had to be carefully controlled in order to get a robust analytical assay. In order to stabilize rMtL, PEG 6000 was added to the enzyme dilution medium. Furthermore, Triton X-100 was included in the enzyme incubation solution.*

*The analytical as well as technical conditions have been optimized, resulting in a method with good precision, sensitivity and speed of analysis. The Michaelis–Menten constant, $K_m$, was determined to be 22 μM syringaldazine. LOQ was determined to be 0.010 $Uml^{-1}$, LOD to be 0.0002 $Uml^{-1}$. The analytical range of the enzyme dilution curve was from 0.01 to 0.044 $Uml^{-1}$. The repeatability was 1.9%, the reproducibility 3.1%. Testing the robustness of the method showed that the most sensible factors in the rMtL analysis in decreasing range were: incubation temperature, concentration of Triton X-100, molarity and pH of the incubation buffer, and finally the concentration of syringaldazine.*

### Introduction

Laccase, p-diphenol: $O_2$ oxidoreductase (EC 1.10.3.2.) rMtL is an extracellular oxidoreductase formed during aerobic fermentation of a soft-rot fungus, the thermophilic ascomycete, *Myceliophthora thermophila* (earlier called *Sporotrichum thermophile*). At Novo Nordisk, the gene responsible for the formation of rMtL was expressed in a strain of *Aspergillus oryzae* using recombinant DNA technique [1, 2]. The rMtL protein is an acidic glycoprotein with an 85 kDa molecular mass. The protein is composed of 572 amino acids and is highly glycosylated between 20 and 40% (mannose, 55%; galactose, 39%; and glucosamine, 6%). Native MtL has a molecular mass of 80 kDa. The difference in molecular mass between rMtL and native MtL is primarily caused by a different degree of glycosylation.

The enzyme is N-terminal blocked with N terminal pyroglutamate residues. pI is 4.2 [3–5]. rMtL has four copper atoms in the active site and a distinctive three-subdomain structure. The rMtL contains one type 1, T1, site and one type 4, T4, trinuclear copper cluster. The T1 site is in domain 3, whereas the trinuclear cluster is at the border between domains 1 and 3 with ligands from each domain. Furthermore, there are three S–S bindings. Copper in laccase enzymes involves inner-sphere electron transfer in binding activation and reduction of, e.g. dioxygen.

Functionally, rMtL couples the four electron reduction of dioxygen to water with the oxidation of a given substrate. The redox potential ($E_0$) for the rMtL laccase is about 465 mV, whereas the laccase from the basidiomycete *Polyporus pinsitus* has an $E_0$ value of about 775 mV. The reason for the very high reduction potentials among fungal laccases remains to be determined [6].

The function of the T1 site within the enzyme is long-range intramolecular electron transfer, shuttling electrons from the substrate to the trinuclear cluster. The electrons are transferred from the T1 copper to the trinuclear cluster approximately 13 Å away along the cysteine–histidine pathway. The trinuclear cluster is the site of dioxygen binding and reduction. There are several main functions of laccases. Degrading lignin different enzymes operate synergistically. Laccases may, e.g. remove potentially toxic phenols produced during lignin degradation [7]. Also, laccases appear to be involved in sporulation [8], pigment production [9] and plant pathogenesis, detoxification [10].

### Reaction mechanism of laccase

Molecular oxygen is chemically inert, and a catalyst, e.g. rMtL, is needed in order to reduce dioxygen. In the determination of the activity of rMtL, molecular oxygen is used as a final electron acceptor. Atmospheric dioxygen is directly reduced to oxygen in two molecules of water during liberation of four electrons without hydrogen peroxide being an intermediate step. Laccases catalyse the oxidation of phenolic compounds, and are found in both plants and fungi [11]. The metabolic pathway of syringic and vanillic acids is to a great extent dependent on the pH of the reaction [12]. The operation mode of a laccase enzyme is, e.g. examined with Rhus species, which in the white sap contain phenols (uristriol, laccal). These phenols are catalysed by laccase, and in the presence of dioxygen oxidized into quinones. Hereby, active radicals are formed, which spontaneously are polymerized, protecting the structure, comparable to the blood clotting system in mammals.

0142-0453/98 $12·00 © 1998 Taylor & Francis Ltd

### Theory

*Choice of substrate chromophore*

rMtL has a low level of specificity towards the reductant. Therefore, many compounds have been used as substrates in the activity determination of laccases, e.g. guajacol [13], 2,4 dimetoxyphenol [14], 2,2-azino-bis(3-ethyl-benzothiazoline-6-sulphonate) (ABTS), ABTS combined with para-hydroxybenzoic acid/$NaN_3$ [15, 16] and syringaldazine [17–20]. Most of these substrates are insoluble in water. This paper represents the work using syringaldazine (pKa = 8.8) as the substrate chromophore [21].

The oxidation of the diphenolic syringaldazine involves the release of two protons. Compared to that, ABTS is a non-phenolic benzothiazoline compound, thus no use as a substrate chromophore detecting laccase activity. The present laccase is defined here as a p-diphenol: $O_2$ oxidoreductase, meaning that at least two phenolic groups in the para position have to be involved in the reaction. Of the given substrates, only syringaldazine fulfils this criterion. Syringaldazine is moderately water soluble, and the reaction product is well defined [22].

The principle of the present method is as follows: under aerobic conditions, rMtL laccase catalyses the oxidation of syringaldazine, forming tetrametoxy-bismethylene quinone. The violet colour thus formed is measured kinetically at 530 nm. For the reaction survey, see figure 1.

*Description of the manual LAMU method*

One LAMU unit is the amount of enzyme capable of converting 1 µmol of syringaldazine per min under the given reaction conditions.

All the reagents used throughout this work were of analytical grade and prepared with water of MilliQ quality. A stock solution was composed of syringaldazine (0.56 mM dissolved in 96% ethanol). Prior to analysis, the syringaldazine was diluted with water to 0.28 mM. In the manual method, 4.0 ml of TRIS buffer, 25 mM, pH 7.5, was preheated for 10 min at 30 °C. Afterwards, 100 µl of enzyme dilution was added under mixing. Finally, 300 µl of syringaldazine (0.28 mM) was added under mixing. The incubation temperature was 30 °C during the kinetic measurement in a cuvette (530 nm). A lag phase occurred in the reaction. Therefore, the difference in absorbance values, $A_{90s}$–$A_{60s}$, was correlated to the enzyme activity. Regarding the calculation, a µmol extinction coefficient at 0.065 of the formed tetraoxy-bismethylene quinone was used [17]. Note that the order in which the different reagents were mixed in the analysis was a critical parameter.

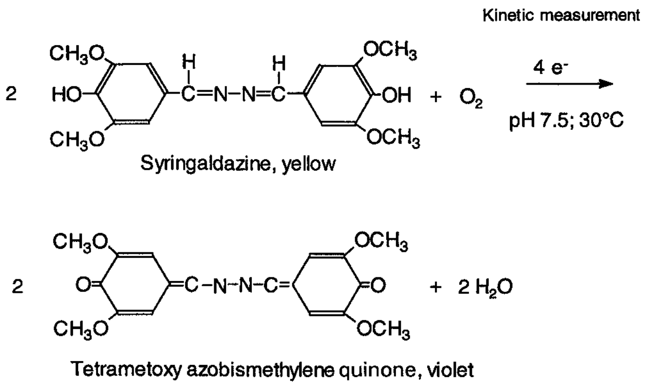

*Figure 1. Overview of the conditions for the rMtL–syringaldazine reaction.*

*Choice of incubation buffer*

Regarding the stability of rMtL, different buffers were tested using routine conditions. The buffers were (0.1 M): TRIS, MOPS, HEPES, TAPS and PIPES. The best choice was TRIS buffer with a satisfying buffer capacity at pH 7–9.

*pH activity*

Using the routine analytical conditions, the optimum of the pH activity curve for a highly purified laccase sample was determined. The pH value was dependent on the type of buffer used as well as on the buffer molarity. If, e.g. a combined buffer of sodium acetate (50 mM) and sodium phosphate (50 mM) was used, the pH maximum was 6.5. Using a combined buffer composed of sodium acetate (25 mM) and TRIS (25 mM), the maximum was pH 7.0. Analysing with a pure TRIS buffer, 25 mM, the pH optimum changed to pH 7.5. This pH value was chosen as a routine condition (see figure 2).

*Temperature activity*

Routine analytical conditions were used. The results are shown in figure 3. The temperature optimum was found to be 70 °C ($t$ = 90 s). As the sensitivity of the syringaldazine reaction was at a satisfying level, 30 °C was preferred as the routine temperature during the reaction.

*Enzyme dilution medium*

In highly diluted solutions, oxidoreductase enzymes in general are unstable, which from an analytical point of view is a critical aspect. An example is peroxidase, where

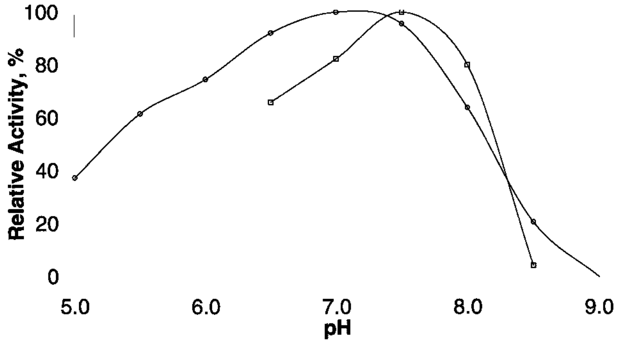

*Figure 2. pH activity curve with a combined buffer composed of sodium acetate (25 mM) and sodium phosphate (25 mM) [○]. pH range: 5.0–9.0, pH activity curve with a pure Tris buffer (25 mM). [□]. pH range: 6.5–8.5.*

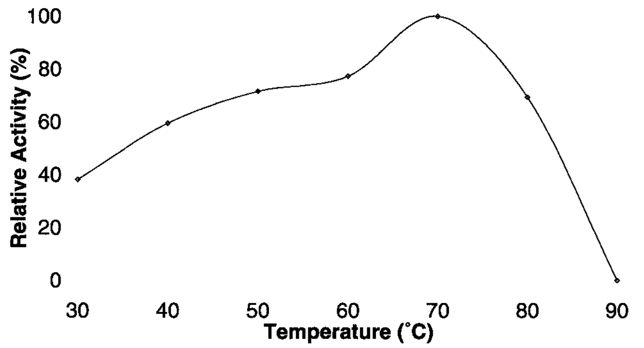

*Figure 3. Temperature activity from 30 °C to 90 °C.*

an addition of Triton X-405 was essential for the stabilization of the enzyme protein [23]. The same consideration might be transferred to rMtL. Therefore, in order to get a stabile and robust analytical method determining the activity of rMtL, stabilization of the laccase enzyme protein was essential prior to the analysis. Different compounds were tested as enzyme dilution medium. PEG 6000, Triton X-100 and bovine serum albumin, respectively, were each prepared in a solution of 1 $gl^{-1}$. The best result was obtained with the PEG 6000.

*Choice of the concentration of PEG 6000*

Different concentrations of PEG 6000 (1–50 $gl^{-1}$) were dissolved in water and tested as the enzyme dilution medium. Routine analytical conditions were used. Addition of PEG, 50 $gl^{-1}$, resulted in the best stability of rMtL. Higher concentrations of PEG were also examined, but no further improvement in the stability of the rMtL was obtained. Therefore, PEG 6000, 50 $gl^{-1}$, was chosen as the routine condition.

*Addition of different salts*

The following salts were successively added to the enzyme dilution medium (50 g of PEG 6000 $l^{-1}$) in order to examine a possible interference on the rMtL assay. Routine analytical conditions were used. Potassium chloride (KCl) 1–200 $gl^{-1}$, cupric sulphate ($CuSO_4$) 10–100 $mgl^{-1}$, magnesium sulphate ($MgSO_4$) 1 $gl^{-1}$ and magnesium chloride ($MgCl_2$) 1 $gl^{-1}$ were added, respectively. With the given concentrations, only the addition of KCl caused a decrease in activity. The following example showed that the chloride ion was responsible for this decreasing effect.

*The effect of sodium chloride*

In order to make a further examination of the chloride effect on the rMtL assay, sodium chloride (NaCl) was introduced to the enzyme dilution medium (PEG 50 $gl^{-1}$) applying different concentrations hereof. The results are shown in table 1. The chloride ion had a very negative influence on the enzyme activity, and a 5% decrease in response was seen by adding only 1 $gl^{-1}$. In order to avoid the chloride ion, the TRIS buffer used was adjusted to pH 7.5 with maleic acid.

*Table 1. Addition of sodium chloride to the enzyme dilution medium.*

| | Relative Activity % |
|---|---|
| Reference | 100 |
| NaCl, 1 $gl^{-1}$ | 95 |
| NaCl, 10 $gl^{-1}$ | 89 |
| NaCl, 100 $gl^{-1}$ | 61 |
| NaCl, 200 $gl^{-1}$ | 59 |

*Table 2. Addition of sulphate or phosphate ions to the enzyme dilution medium*

| | Relative Activity % |
|---|---|
| Reference | 100 |
| $Na_2SO_4$, 0.5 $gl^{-1}$ | 99 |
| $Na_2SO_4$, 1 $gl^{-1}$ | 101 |
| $Na_2SO_4$, 10 $gl^{-1}$ | 98 |
| $Na_2SO_4$, 100 $gl^{-1}$ | 90 |
| $Na_2SO_4$, 200 $gl^{-1}$ | 80 |
| $Na_2HPO_4$, 0.5 $gl^{-1}$ | 100 |
| $Na_2HPO_4$, 1 $gl^{-1}$ | 95 |
| $Na_2HPO_4$, 10 $gl^{-1}$ | 94 |
| $Na_2HPO_4$, 50 $gl^{-1}$ | 83 |
| $Na_2HPO_4$, 100 $gl^{-1}$ | 76 |

*The effect of sulphate or phosphate ions*

Different concentrations of sulphate or phosphate ions were added to the dilution medium (PEG 50 $gl^{-1}$) testing the influence on the stability of the rMtL. The obtained results are shown in table 2. Both sulphate and phosphate ions seemed to have a negative effect on the rMtL, and should be avoided in concentrations higher than 10 $gl^{-1}$.

*Addition of Triton X-100*

Addition of Triton X-100 to the enzyme dilution medium and/or to the incubation buffer was also tried. The best result was achieved by adding 10 g of Triton X-100 $l^{-1}$ to the enzyme dilution medium (in addition to the routinely used 50 g of PEG 6000 $l^{-1}$). Addition of Triton X-100 to the incubation buffer did not improve the stabilization during kinetic measurement.

*Conclusion: Manual method*

A method determining the activity of rMtL was developed with the following conditions:

| | |
|---|---|
| Incubation buffer | 4.0 ml of TRIS buffer, pH 7.5, 25 mM |
| Enzyme dilution medium | 50 g of PEG 6000 $l^{-1}$ and 10 g of Triton X-100 $l^{-1}$ dissolved in water. |
| Sample volume | 100 µl (enzyme activity range: 0.07–0.28 $LAMUml^{-1}$) |
| Substrate chromophore | Syringaldazine 0.28 mM (dissolved in 48% ethanol) |

| | |
|---|---|
| Syringaldazine-R volume | 300 µl |
| Incubation temperature | 30 °C |
| Wavelength | 530 nm |
| Kinetic measuring from | 60–90 s |
| Calculation | $t_{90\,s}$–$t_{60\,s}$ (linear part of the kinetic curve) |

### The automated (COBAS) FARA method

### *Experimental*

After development of the manual method determining the activity of rMtL, an automated Cobas Fara method was evaluated using the results obtained in the development of the manual method.

### *Apparatus*

An automated centrifugal analyser, a Cobas Fara from Hofmann–La Roche, Basel, Switzerland, was used. This instrument was well suited for kinetic measurements, having a great capacity for analyses.

### *Reagents*

All the reagents used were of analytical grade. The water used was of MilliQ water quality. TRIS buffer stock solution, 1 M, 121.1 g of TRIS (hydroxymethyl) aminomethane (Sigma, USA) was dissolved in 1 l of water.

Maleic acid (Merck 800380, Germany) was prepared by dissolving 23.2 g in 200 ml of water. Triton X-100, stock solution 10%, prepared by dissolving 25.0 g in 250 ml of water. TRIS buffer, 25 mM, pH 7.5. Application solution, 12.5 ml of TRIS buffer stock solution, 5.0 ml of maleic acid stock solution, 2.5 ml of Triton X-100 stock solution and water up to 500 ml.

Enzyme dilution medium, 50.0 g of PEG 6000 (Merck, Germany) was dissolved in 1 l of water.

Syringaldazine stock solution, 0.56 mM, 10.0 mg of syringaldazine (S-7896, Sigma, USA) was dissolved in 96% ethanol (Danisco, Denmark). Stability was one month at −18 °C. Syringaldazine: solution for application, 0.22 mM, 4.0 ml of the syringaldazine stock solution and made up to 10 ml with water.

Check for the syringaldazine reagent: 2.0 ml of 0.22 mM syringaldazine reagent, 4.0 ml of 96% ethanol and made up to 10.0 ml with water. The absorbance of this solution should be about 1.8 read at 360 nm (against 6% ethanol).

### *Samples*

A highly refined rMtL preparation was used throughout this work. The preparation was refined at Novo Nordisk (2019 LAMUg$^{-1}$). Fermentation sample: the process sample from Novo Nordisk was used.

### *Procedure*

The samples were diluted to approx. 0.025 LAMUml$^{-1}$. The dilution medium was 50 g of PEG 6000 dissolved in 1 l of water. Due to an enzyme activation phase, the diluted samples had to stand for at least 15 min before analysing on the Cobas Fara analyser. The working area ranged from 0.010 to 0.044 LAMUml$^{-1}$.

Reagents and samples were placed in racks before being dispensed by the pipette tool of the Cobas Fara, respectively. The sample volume was 25 µl, water 20 µl and buffer volume 325 µl. The rotor with the cuvettes started to accelerate, thus mixing the volumes. Finally, 30 µl of syringaldazine was pipetted into the small compartment of the rotor. When the incubation temperature had reached its level, the centrifugation started, and the absorbance readings took place. A total of 25 absorbance values was read from each curette with an interval of 5 s. Rectilinear kinetic measurements obtained between the 12th and 24th reading were used for the calculation, and the absorbance per minute was calculated for each sample.

### *Calculation*

Unit definition: as already defined, 1 LAMU unit is the amount of enzyme capable of converting 1 µmol syringaldazine per min under the given reaction conditions.

$$(A \times \mathrm{Vol} \times D) \times W^{-1} = \mathrm{LAMU\,g^{-1}}$$

$$A = F \times \mathrm{ABS} \times \mathrm{min}^{-1}(\mathrm{LAMU\,g^{-1}})$$

where: Vol = sample dilution volume (ml); $D$ = further dilution of the sample (ml ml$^{-1}$); $W$ = weight of the sample in work (g).

$$F = V \times 10^{-3} \times (v \times E \times b)^{-1}$$

where: $V$ = total volume in the reaction solution (ml); $v$ = sample volume in the reaction solution (ml); $E$ = µmolar extinction coefficient at 530 nm (0.065 per 1 cm light path); $b$ = light path. The total volume in the reaction is 400 µl. That volume corresponds to a light path in the cuvette at 1.6 cm; $10^{-3}$ is µmol l$^{-1}$ converted to µmol ml$^{-1}$.

### *Addition of Triton X-100 to the incubation buffer*

In order to avoid adhesion problems in the Cobas Fara system, Triton X-100 was added to the incubation buffer. Two concentrations of Triton X-100 (0.5 and 10 g l$^{-1}$) were added to the incubation buffer (TRIS, 25 mM, pH 7.5) using 50 g of PEG 6000 l$^{-1}$ dissolved in water as the enzyme dilution medium. The best result was obtained with 0.5 g of Triton X-100 l$^{-1}$. Addition of higher concentrations of Triton X-100 was really a critical parameter, e.g. using 10 gl$^{-1}$ hereof gave a residual laccase activity of less than 50%.

### *Stabilization of rMtL in the enzyme dilution medium*

Various concentrations of PEG 6000 were used as enzyme dilution media: 1, 10, 25 and 50 gl$^{-1}$. The best enzyme stability and linearity of the enzyme standard curve were found using 50 g of PEG 6000 l$^{-1}$ without any addition of Triton X-100 to the enzyme dilution medium.

*Activation of rMtL*

Using the routine analytical conditions, the activation of the rMtL in the final sample dilution was examined. A highly refined sample as well as a fermentation sample were analysed immediately after the dilution (5 min), and reanalysed after 14, 25, 45 and 60 min, respectively. The activity increased slightly, about 2%, within the first 10 min. Consequently, the diluted rMtL solutions had to be activated for at least 15 min before analysis.

*Determination of the Michaelis–Menten constant* ($K_m$)

Regarding the given reaction to be either a reaction of 0 order or pseudo 1st order, the $K_m$ was determined. The sample was diluted in two levels and analysed at two different concentrations of the substrate (0.28 and 0.14 mM). Using Hanes plot [24], the average $K_m$ value was found to be 22 μM, which shows a high degree of affinity between rMtL and syringaldazine. Therefore, the actual substrate concentration was found to be at a satisfying level 10 times higher than the $K_m$ value.

*Enzyme working range*

The working range was determined to be from 0.010 to 0.044 LAMUml$^{-1}$.

*Precision, LOD and LOQ*

The precision of the present method [25] was examined within a day expressed by repeatability and between days by the reproducibility. The repeatability for a given sample analysing five levels each with six separate dilutions was 1.9%. The reproducibility for a sample also analysed at four levels with two separate dilutions over six days was found to 3.1%. The limit of detection (LOD) and limit of quantitation (LOQ) were calculated to be 0.0002 LAMUml$^{-1}$ and 0.010 LAMUml$^{-1}$, respectively.

*Robustness of the method*

Testing the robustness of the method [25] showed that many parameters were important re stabilization of the analytical conditions. The most critical ones were, in decreasing order: incubation temperature, concentration of Triton X-100, molarity and pH of the incubation buffer, and the concentration of syringaldazine. The results were validated according to Youden's test [26].

*Conclusion, Cobas Fara method*

A Cobas Fara method determining the activity of rMtL was developed. The following analytical conditions were chosen:

| | |
|---|---|
| Incubation buffer | TRIS buffer 25 mM, pH 7.5, 0.5 g of Triton X-100 l$^{-1}$. |
| Enzyme diluent | 50 g of PEG l$^{-1}$ |
| Substrate chromophore | Syringaldazine 0.22 mM |
| Incubation temperature | 30 °C |
| Wavelength | 530 nm |
| Kinetic measurement | from 60 to 120 s |

From an analytical point of view, rMtL was a very delicate oxidoreductase, where many factors had to be under control in order to get a robust analytical assay.

Stability of the diluted sample: >8 h at 22 °C.

Stability of the syringaldazine application reagent: 2 h at 22 °C.

### Summary

Determining the LAMU activity, an automated and fast routine method using a Cobas Fara analyser was developed. The method is sensitive with good precision. The type of samples may either be fermentation samples or highly purified samples. Analysing 30 samples, the analytical time from sample uptake to appearance of recorder response is about 15 min.

### References

1. Christensen, T., Wøldike, H., Boel, E., Mortensen, S. B., Hjortshøj, K., Thim, L. and Hansen, M. T., 1988, *Biotechnology*, **6**, 1419.
2. Christensen, T., 1994, Application: Aspergillus oryzae as host for production of industrial enzymes. *FEMS Symp.*, **69**, 251.
3. Yaver, D. S., Xu, F. and Schneider, P., 1996, *Appl. Environ. Microb.*, **62**, 834.
4. Mondorf, K., 1994, *NOVO Nordisk Report.*
5. Berka, R. M., Schneider, P., Golightly, E. J. and Brown, S. H., 1997, *Appl. Environ. Microb.*, **6**, 3151.
6. Solomon, E. I., Sundaram, U. M. and Machokin, T. E., 1996, *Chem. Rev.*, 2563.
7. Youn, H.-D., Hah, Y. C. and Kang, S.-O., 1995, *FEMS Microbiology Letters*, **132**, 183.
8. Leathan, G. and Stohman, M. A., 1981, *J. Gen. Microbiol.*, **125**, 147.
9. Tanaka, C., Tajima, S. and Furusava, M., 1992, *Mycol. Res.*, **96**, 959.
10. Marbach, I., Harel, E. and Mayer, A. M., 1985, *Phytochemistry*, **24**, 2559.
11. Mayer, A. M., 1987, *Phytochemistry*, **26**, 11.
12. Leonowicz, A., Edehill, R. U. and Bollag, J.-M., 1984, *Arch. Microbiol.*, **137**, 89.
13. Zuoari, N., Romette, J. L. and Thomas, D., 1987, *Appl. Biochem. Biotechnol.*, **15**, 213.
14. Shuttleworth, K. L. and Bollag, J.-M., 1986, *Enzyme Microb. Technol.*, **8**, 171.
15. Shin, T., Muran, S. and Matsumura, E., 1987, *Anal. Biochem.*, **166**, 380.
16. Mutao, S., 1985, *J. Pharm. Society of Japan*, **105**, 86.
17. Bauer, R. and Rupe, C. O., 1971, *Anal. Chem.*, **43**, 421.
18. Petroski, R.J., Peczynska-Czoch, W. and Rosazza, J. P., 1980, *Appl. Environ. Microb.*, **40**, 1003.
19. Leonowics, A. and Grzyknowicsz, K., 1981, *Enzyme Microb. Technol.*, **3**, 55.
20. Ander, P. and Eriksson, K.-L., 1987, *Biotech. Appl. Biochem.*, **9**, 160.
21. Hapiot, P. and Pinson, J., 1993, *J. Electroanal. Chem.*, **353**, 225.
22. Wahleithner, J .A., Xu, F., Brown, K. M. and Brown, S. H., 1996, *Curr. Genet.*, **29**, 3.
23. Holm, K. A., 1995, *Analyst*, **120**, 2101.
24. Hanes, C. S., 1932, *Biochem. J.*, **26**, 1406.
25. Holm, K. A., 1996, Novo Nordisk validation report re LAMU assay.
26. Youden, W. J. and Steiner, E. H., 1975, Statistical manual of the association of official analytical chemist. *AOAC.*

